# Iron Limitation in *Klebsiella pneumoniae* Defines New Roles for Lon Protease in Homeostasis and Degradation by Quantitative Proteomics

**DOI:** 10.3389/fmicb.2020.00546

**Published:** 2020-04-24

**Authors:** Benjamin Muselius, Arjun Sukumaran, Jason Yeung, Jennifer Geddes-McAlister

**Affiliations:** Department of Molecular and Cellular Biology, University of Guelph, Guelph, ON, Canada

**Keywords:** quantitative proteomics, iron limitation, *Klebsiella pneumoniae*, secretome, Lon protease

## Abstract

Nutrient adaptation is key in limiting environments for the promotion of microbial growth and survival. In microbial systems, iron is an essential component for many cellular processes, and bioavailability varies greatly among different conditions. In the bacterium, *Klebsiella pneumoniae*, the impact of iron limitation is known to alter transcriptional expression of iron-acquisition pathways and influence secretion of iron-binding siderophores, however, a comprehensive view of iron limitation at the protein level remains to be defined. Here, we apply a mass-spectrometry-based quantitative proteomics strategy to profile the global impact of iron limitation on the cellular proteome and extracellular environment (secretome) of *K. pneumoniae*. Our data define the impact of iron on proteins involved in transcriptional regulation and emphasize the modulation of a vast array of proteins associated with iron acquisition, transport, and binding. We also identify proteins in the extracellular environment associated with conventional and non-conventional modes of secretion, as well as vesicle release. In particular, we demonstrate a new role for Lon protease in promoting iron homeostasis outside of the cell. Characterization of a Lon protease mutant in *K. pneumoniae* validates roles in bacterial growth, cell division, and virulence, and uncovers novel degradation candidates of Lon protease associated with improved iron utilization strategies in the absence of the enzyme. Overall, we provide evidence of unique connections between Lon and iron in a bacterial system and suggest a new role for Lon protease in the extracellular environment during nutrient limitation.

## Introduction

Adaptation to nutrient limitation is a key determinant of microbial growth and fitness in medical, environmental, and industrial contexts. For example, iron is an essential nutrient for bacterial growth, and many naturally occurring environments have low iron bioavailability (Andrews et al., 2003). In bacteria, iron bioavailability is implicated in many cellular processes, including DNA replication, metabolism, and the response to oxidative stress (Abdul-Tehrani et al., 1999; Posey and Gherardini, 2000; Andrews et al., 2003). Within an organism, the levels of available iron are tightly regulated to promote cellular function while limiting cellular damage associated with the production of free radicals (Skaar, 2010). To acquire iron from the environment, bacteria have adapted several strategies. These include the use of high-affinity iron-capture methods, such as siderophores, which are secreted and possess iron-binding properties (Miethke and Marahiel, 2007; Holden and Bachman, 2015; Holden et al., 2016, 2018). In addition, high-affinity membrane transporters transport iron into the interior of the cell and transfer it to iron-trafficking proteins (Faraldo-Gómez and Sansom, 2003; Wandersman and Delepelaire, 2004; Miethke and Marahiel, 2007; Skaar, 2010; Sheldon et al., 2016). In *Klebsiella pneumoniae*, a Gram-negative, opportunistic pathogen, iron availability influences the transcriptional expression of iron-acquisition pathways and influences the secretion of iron-binding siderophores (Holden et al., 2018). These siderophores have demonstrated roles in the induction of inflammation, bacterial dissemination, and activation of hypoxia (Holden et al., 2016).

To adapt under nutrient-limited conditions, bacteria often induce protein-level changes through transcriptional regulation and protease-mediated protein degradation. Proteases are responsible for the degradation of misfolded or defective proteins, as well as the regulation of stress-related proteins, labile regulators, and chaperones (Gur and Sauer, 2008). These activities support their roles in global control of cellular activities related to metabolism, stress, virulence, and antibiotic resistance (Gottesman, 1996). For example, the ATP-dependent, cytoplasmic serine protease, Lon protease, plays important roles in Gram-negative bacteria. In *Escherichia coli*, Lon protease is well characterized and regulates cellular homeostasis through degradation of abnormal proteins, and degrades physiological targets, including the antitermination protein of phage, the cell division inhibitor SulA, and the capsule transcriptional activator RcsA (Mizusawa and Gottesman, 1983; Maurizi, 1987; Torres-Cabassa and Gottesman, 1987; Kobiler et al., 2004; Nazir and Harinarayanan, 2016). In *Pseudomonas aeruginosa*, Lon is important for controlling virulence, antibiotic resistance, and stress response (Kindrachuk et al., 2011; Fernández et al., 2012). In addition, *lon* mutants show increased susceptibility to fluoroquinolones due to reduced ability of the cell to trigger a DNA-damage response and an elongated (filamentous) morphology likely associated with accumulation of SulA (Brazas et al., 2007; Breidenstein et al., 2008, 2012a). Recently, quantitative proteomics uncovered a novel proteolytic target of Lon, the RNA-binding protein Hfq, which implicates a regulatory cascade controlling multiple phenotypes via posttranscriptional regulation by small non-coding RNAs (sRNAs) (Fernandez et al., 2016). Moreover, integrated proteomic profiling defined novel substrates and functions of Lon protease in *E. coli* using a Lon trapping variant to translocate substrates, revealing 14 new potential targets with diverse roles in sulfur assimilation, nucleotide biosynthesis, and central energy metabolism (Arends et al., 2018).

A connection between iron homeostasis and Lon protease has been explored in microbial systems, including *Salmonella*, where Lon protease degradation of FeoC, a component of the Feo system, is responsible for ferrous ion import under altered oxygen conditions (Kim et al., 2015). In addition, work in *Saccharomyces cerevisiae* describes Lon protease degradation of Isu, a mitochondrial scaffold protein involved in assembly of iron–sulfur clusters, demonstrating a dynamic interplay between Lon protease and protein factors throughout the Fe–S cluster assembly and transfer process (Song et al., 2012). Moreover, detection of bacterioferritin as a potential Lon substrate supports this association (Arends et al., 2018). However, a connection between iron availability and Lon protease production in *K. pneumoniae* has yet be defined. Such an investigation is critical to comprehensively profile the fundamental processes underscoring the linkage between iron homeostasis, Lon protease degradation, and bacterial survival.

One of the most powerful techniques to explore the global impact of nutrient limitation on cellular processes is mass-spectrometry-based quantitative proteomics. This technology provides unbiased, robust, and sensitive measurements of protein abundance to define comprehensive cellular proteomes and secretomes (the extracellular environment), as well as protein modifications and interaction networks (Aebersold and Mann, 2016). In the present study, we use state-of-the-art mass spectrometry-based quantitative proteomics [liquid chromatography with tandem mass spectrometry (LC-MS/MS)] to profile the cellular proteome and secretome of *K. pneumoniae* in response to iron limitation. Our data defines the deepest proteome of *K. pneumoniae* to date, supporting our observations of the global reprogramming of cellular processes under iron-limited conditions. Specifically, we report an important impact on transcriptional regulation and an emphasis on proteins associated with iron acquisition, transport, and binding. These data achieve detection of proteins directly and indirectly involved in maintaining iron homeostasis within a bacterial system. We also define the presence of proteins in the extracellular environment associated with conventional and non-conventional modes of secretion and vesicle release. In particular, we demonstrate a significant increase in abundance of Lon protease in the secretome during iron limitation, suggesting a new role for the protease associated with influencing iron homeostasis outside the cell. With this information, we characterize the role of Lon protease in *K. pneumoniae* related to bacterial growth, cell division, and virulence, as well as uncover novel degradation candidates and relate accumulating protein abundance to improved iron utilization strategies in the absence of the protease. In summary, we propose a unique role for Lon protease in the extracellular environment of *K. pneumoniae* during nutrient limitation and provide evidence of novel connections between Lon and iron in bacterial systems.

## Materials and Methods

### Bacterial Strains, Growth Conditions, and Media Preparation

*K. pneumoniae* wild type (WT) (K52 serotype) was maintained on Tryptic Soy Broth (TSB) (Fluka Analytical) agar plates. Low iron media (LIM) was prepared as previously described (Holden et al., 2018). Briefly, M9 minimal media was prepared with Chelex® 100-treated (Bio-Rad) dH_2_O and supplemented with 10 or 100 μM Fe_2_(SO_4_)_3_ for replete conditions. For *in vitro* cultures, *K. pneumoniae* was grown overnight at 37°C in TSB, washed twice with LIM, and subcultured in LIM or LIM + 10 or 100 μM Fe_2_(SO_4_)_3_ and grown to mid-log phase. For macrophage infection, *K. pneumoniae* was grown overnight at 37°C in TSB, subcultured in TSB to mid-log phase. Samples were collected in triplicate for phenotypic and macrophage infection assays and collected in quadruplicate for the proteomic analyses.

### Sample Preparation for LC-MS/MS Analysis

Cellular proteome samples were processed in quadruplicate per condition and subjected to a modified total proteome extraction protocol (Ball and Geddes-McAlister, 2019). Briefly, samples were washed twice with phosphate-buffered saline (PBS) and resuspended in 100 mM Tris–HCl (pH 8.5) containing a protease inhibitor cocktail tablet. Using a probe sonicator (Thermo Fisher Scientific), samples were lysed in an ice bath for three cycles (30% power, 30 s on/30 s off), and 2% (final) sodium dodecyl sulphate (SDS) and 10 mM dithiothreitol (DTT) were added, followed by incubation at 95°C for 10 min with shaking at 800 rpm. The samples were cooled, 55 mM iodoacetamide (IAA) was added, incubated at room temperature for 20 min in the dark, and then, 100% ice cold acetone (final concentration of 80%) was added prior to storage at −20°C overnight. Samples were collected by centrifugation at 13,500 rpm at 4°C for 10 min, washed twice with 80% acetone, and air dried. Pellets were resolubilized in 8 M urea/40 mM HEPES and a bovine serum albumin (BSA) tryptophan assay determined protein concentrations (Wisniewski and Gaugaz, 2015). Samples were diluted in 50 mM ammonium bicarbonate and digested overnight with a mixture of LysC and trypsin proteases (Promega, protein/enzyme ratio, 50:1). Digestion was stopped with 10% *v*/*v* trifluoroacetic acid (TFA), and 50 μg of the acidified peptides was loaded onto STop And Go Extraction (STAGE) tips (consisting of three layers of C18) to desalt and purify according to the standard protocol (Rappsilber et al., 2007).

Secretome samples were processed in quadruplicate per condition and subjected to an in-solution digestion as previously described (Geddes-McAlister and Gadjeva, 2019). Culture supernatant was filtered through 0.22 μm syringe filters to remove any cellular debris prior to processing. Briefly, one-third sample volume of 8 M urea/40 mM HEPES was added to filtered supernatant followed by vortexing. Samples were reduced with 10 mM DTT, alkylated with 55 mM IAA, followed by enzymatic digestion and STAGE-tip purification as described above.

### LC-MS/MS

Samples were eluted from STAGE-tips with 50 μl buffer B [80% acetonitrile (ACN) and 0.5% acetic acid], dried, and resuspended in 12 μl buffer A^∗^ (0.1% TFA). Six microliters of each sample was analyzed by nanoflow liquid chromatography on an Ultimate 3000 LC system (Thermo Fisher Scientific) online coupled to a Fusion Lumos Tribrid mass spectrometer (Thermo Fisher Scientific) through a nanoelectrospray flex-ion source (Thermo Fisher Scientific). Samples were loaded onto a 5 mm μ-precolumn (Thermo Fisher Scientific) with 300 μm inner diameter filled with 5 μm C18 PepMap100 beads. Peptides were separated on a 15 cm column with 75 μm inner diameter with 2 μm reverse-phase silica beads and directly electrosprayed into the mass spectrometer using a linear gradient from 4 to 30% ACN in 0.1% formic acid over 45 min at a constant flow of 300 nl/min. The linear gradient was followed by a washout with up to 95% ACN to clean the column followed by an equilibration stage to prepare the column for the next run. The Fusion Lumos was operated in data-dependent mode, switching automatically between one full scan and subsequent MS/MS scans of the most abundant peaks with a cycle time of 3 s. Full-scan MS1s were acquired in the Orbitrap analyzer with a resolution of 120,000, scan range of 400–1,600 *m*/*z*. The maximum injection time was set to 50 ms with an automatic gain control target of 4e5. The fragment ion scan was done in the Orbitrap using a quadrupole isolation window of 1.6 *m*/*z* and higher-energy collisional dissociation (HCD) fragmentation energy of 30 eV. Orbitrap resolution was set to 30,000 with a maximum ion injection time of 50 ms and an automatic gain control target set to 5e4.

### Raw Data Processing

Raw files were analyzed together using MaxQuant software (version 1.6.0.26.) (Cox and Mann, 2008). The derived peak list was searched with the built-in Andromeda search engine against the reference *K. pneumoniae* subsp. *pneumoniae* ATCC 700721 proteome (August 2018; 5,127 sequences) from Uniprot (Cox et al., 2011)^1^. The parameters were as follows: strict trypsin specificity, allowing up to two missed cleavages; minimum peptide length was seven amino acids; carbamidomethylation of cysteine was a fixed modification; and *N*-acetylation of proteins and oxidation of methionine were set as variable modifications. A minimum of two peptides required for protein identification and peptide spectral matches were filtered using a target-decoy approach at a false discovery rate (FDR) of 1%. “Match between runs” was enabled with a match time window of 0.7 min and an alignment time window of 20 min (Cox et al., 2014). Relative, label-free quantification (LFQ) of proteins used the MaxLFQ algorithm integrated into MaxQuant using a minimum ratio count of one (Cox et al., 2014). The mass spectrometry proteomics data have been deposited in the PRIDE partner repository for the ProteomeXchange Consortium with the data set identifier: PXD015623.

### Bioinformatics

Further analysis of the MaxQuant-processed data (“proteingroups.txt” file) was performed using Perseus (version 1.6.2.2) (Tyanova et al., 2016). Hits to the reverse database, contaminants, and proteins only identified with modified peptides were eliminated. LFQ intensities were converted to a log scale (log_2_), and only those proteins present in triplicate within at least one sample set were used for further statistical processing (valid-value filter of 3 in at least one group). Missing values were imputed from a normal distribution (downshift of 1.8 standard deviations and a width of 0.3 standard deviations). A Student’s *t-*test identified proteins with significant changes in abundance (*p* ≤ 0.05) with multiple hypothesis testing correction using the Benjamini–Hochberg FDR cutoff at 0.05. A principal component analysis (PCA) was performed, as well as a Pearson correlation with hierarchical clustering by Euclidean distance to determine replicate reproducibility, and a Student’s *t* test for 1D annotation enrichment (FDR = 0.05) allowed for visualization of enrichment by gene ontology and keywords within the RStudio platform (R Foundation for Statistical Computing, 2018)^2^. The STRING: Functional Protein Association Networks provided visualization of protein networks (Szklarczyk et al., 2019)^3^.

### Deletion Strain Construction

Mutants were constructed in the *K. pneumoniae* WT (K52 serotype) strain using the lambda red recombinase method (Datsenko and Wanner, 2000). Briefly, oligos were designed for homologous recombination of the KPN_00401 (Lon protease) gene and used to amplify the chloramphenicol-resistant cassette from pKD3 as previously described (Figueira et al., 2013). The WT strain was transformed with the pKD46 (i.e., arabinose-inducible lambda red recombinase) at 30°C by electroporation (Datsenko and Wanner, 2000). This was followed by transformation of WT pKD46 with the *lon* PCR product and plating on TSB + Cm^17^ plates. Colonies were cured of the pKD46 plasmid and confirmed for resistance cassette insertion into the gene of interest by colony PCR. At least two independent mutants were generated. All primer sequences used in this study are provided (Supplementary Table S1).

### Complement Strain Construction

To produce a complement strain for Δ*lon*, Lon protease was amplified from *K. pneumoniae* genomic DNA (gDNA) using primers to produce a full-length *lon* with a His tag upon translation (Supplementary Table S1). PCR amplicon was restriction digested using *Bsp*HI and *Xba*I and ligated into pBAD24, containing an arabinose-inducible promoter, which was digested using *Nco*I and *Xba*I (New England Biolabs). The construct was transformed into *E. coli* TOP10 chemically competent cells via heat shock and screened using colony PCR and restriction enzyme digest (Supplementary Table S1). Recombinant plasmid was propagated in *E. coli* prior to transformation into *K. pneumoniae* Δ*lon* strain. Complementation was activated in the presence of 0.1% arabinose, and confirmation of protein production was done by Western blot and phenotypic assays, as outlined below.

### Western Blot

Whole-cell extracts from *K. pneumoniae* Δ*lon*:LON and an empty vector strain were separated by sodium dodecyl sulfate–polyacrylamide gel electrophoresis (SDS-PAGE) and transferred to a nitrocellulose membrane using a transfer apparatus according to the manufacturer’s protocols (Bio-Rad). After blocking with 3% BSA in 1 × TBS (50 mM Tris, 150 mM NaCl, pH 7.5) overnight at 4°C, the membrane was washed five times with TBST (1 × TBS, 0.05% Tween-20) and incubated with 6xHis Monoclonal Antibody (Takara Bio, United States) for 1 h. Membranes were washed three times for 5 min and incubated with 1:5,000 dilution of antimouse immunoglobulin G (IgG)-alkaline phosphatase secondary antibody (Sigma-Aldrich) for 1 h. Blots were washed with TBST three times and developed using an alkaline phosphate (AP) substrate buffer (1 M Tris, pH 9.5, 5 M NaCl, 1 M MgCl_2_, 50 mg/ml 5-bromo-4-chloro-3-indolyl phosphate, and 5% nitrotetrazolium blue chloride). The experiment was performed in biological and technical duplicates.

### Growth Curves and Microscopy

To examine differences in growth and cell morphology in the *K. pneumoniae* WT, Δ*lon*:LON, and Δ*lon* strains, cells were pregrown in TSB at 37°C overnight, collected by centrifugation at 3,500 rpm × 5 min, washed twice with LIM, and resuspended in LIM. Subcultures were inoculated at 1/100 dilution into LIM, LIM supplemented with 10 μM Fe_2_(SO_4_)_3_, or LIM supplemented with 100 μM Fe_2_(SO_4_)_3_ and incubated at 37°C with shaking. For induction of LON in the complemented strain, 0.1% arabinose was added to each of the growth conditions. For growth curves, OD_600_ measurements were collected every 2 h until stationary phase and repeated on BioTek HM1 plate reader with OD_600_ readings every 15 min. For imaging, four μl of culture was collected, and the capsule was stained with four μl of India ink and examined with differential interference contrast microscopy.

### Iron Utilization Assay

To examine the effects of iron limitation and iron repletion on the *K. pneumoniae* WT, Δ*lon*:LON, and Δ*lon* strains, cells were pregrown in TSB at 37°C, harvested, and diluted in LIM. Tenfold serial dilutions of 1 × 10^8^ cells/ml were plated on LIM medium or LIM supplemented with 10 or 100 μM of Fe_2_(SO_4_)_3_ and incubated at 37°C for 72 h with imaging every 24 h. For induction of LON in the complemented strain, 0.1% arabinose was added to each of the growth conditions. The experiment was performed on two independent mutants of Lon protease (KPN_00401) in biological and technical duplicates.

### Macrophage Infection

Infections were performed as previously described with the following modifications (Cano et al., 2015). BALB/c WT immortalized macrophages were seeded in 12-well plates at 0.1 × 10^6^ cells/well in complete Dulbecco’s modified Eagle’s medium (DMEM) (Glutamax DMEM, 10% FBS, 2 mM Glutamax, 1% sodium pyruvate, 1% L-glutamine, 5% pen/strep) at 37°C, 5% CO_2_. After 48 h, cells were at 70–80% confluence (i.e., 0.5 × 10^6^ cells/well), and fresh media without pen/strep were added. Bacterial cells from WT, Δ*lon*:LON, and Δ*lon* strains were grown in TSB (supplemented with 0.1% arabinose) to mid-log phase (OD_600_ = 1.0), centrifuged at 3,500 rpm for 5 min, and resuspended in complete DMEM (without pen/strep). Cells were counted, and macrophage were infected at a multiplicity of infection (MOI) of 50:1 for 90 min at 37°C, 5% CO_2_. Subsequently, cells were washed with PBS and incubated at 37°C, 5% CO_2_ in growth medium containing 300 μg/ml gentamycin for 90 min. Medium was replaced to decrease the gentamycin concentration to 100 μg/ml for collection at later time points.

### Cytotoxicity Assays

Culture supernatants from *K. pneumoniae*-infected BALB/c macrophages were collected at 1, 3, 6, and 18 h post-inoculation (hpi) for quantification of cell death. Cytotoxicity was quantified colorimetrically with the CytoTox96 lactate dehydrogenase (LDH)-release kit (Promega). The percentage of cytotoxicity was calculated with the formula: 100 × [(experimental release)/(total release + spontaneous release)], in which spontaneous release is the amount of LDH activity in the supernatant of uninfected cells and total release is the activity in macrophage lysates. The cytotoxicity assay was performed in biological triplicate, and the experiment was performed in duplicate.

### Association, Invasion, and Replication/Survival Assay

Following the *K. pneumoniae* infection process outlined above using BALB/c macrophages with WT, Δ*lon*:LON, and Δ*lon* strains, an association, invasion, and replication assay was performed (Wu et al., 2014). Briefly, cultures were collected prior to gentamicin treatment for “association” counting, 1 h hpi for “invasion” counting, and 24 hpi for “replication” counting. Bacterial colony forming units were normalized to number of live macrophage cells present at the time of collection for each strain and time point, and then normalized to the WT. This assay was performed in biological triplicate and the experiment was performed in duplicate.

## Results

### Overview of Iron Limitation in *K. pneumoniae*

**FIGURE 1:**
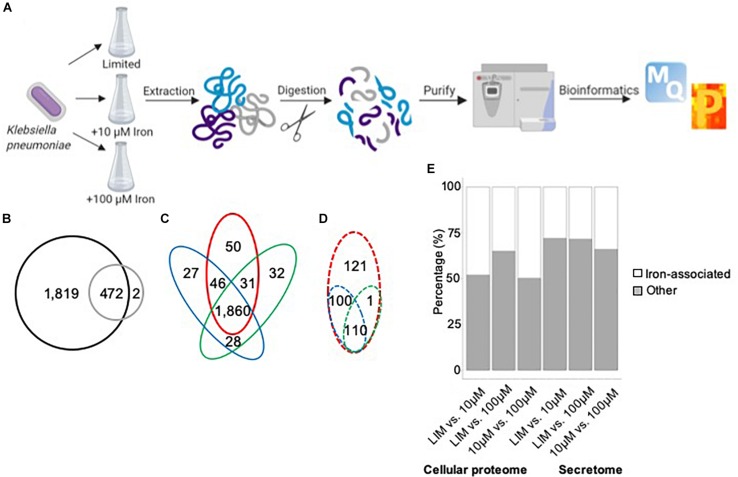
Mass-spectrometry-based proteomics of *K. pneumoniae* during iron limitation. **(A)** Bottom–up proteomics workflow for profiling the cellular proteome and secretome of *K. pneumoniae* under iron-limited (limited) and iron-replete (10 and 100 μM iron) conditions. Proteins were extracted from cells and supernatant followed by enzymatic digestion and purification on C18 resin tips (Rappsilber et al., 2007; Geddes-McAlister and Gadjeva, 2019). The data are processed and analyzed using the publicly available MaxQuant and Perseus platforms (Cox and Mann, 2008; Tyanova et al., 2016). Figure generated with Biorender.com. **(B)** Venn diagram comparing total number of proteins identified in cellular proteome (black) and secretome (gray). **(C)** Venn diagram comparing proteins identified in the cellular proteome under limited (red), 10 μM iron (blue), and 100 μM iron (green) conditions. **(D)** Venn diagram comparing proteins identified in the secretome under limited (red), 10 μM iron (blue), and 100 μM iron (green) conditions. **(E)** Stacked bar plot quantifying the number of iron-associated proteins identified across the conditions in both the cellular proteome and secretome among significantly different proteins [Student’s *t-*test *p-*value < 0.05; false discovery rate (FDR) = 0.05; S0 = 1].

Our proteomic profiling workflow reports the deepest proteome of *K. pneumoniae* to date, with 2,293 proteins identified from 5,126 open reading frames, representing 45% of the proteome (Figure 1A). A comparison between the proteins identified in the cellular proteome vs. secretome shows the majority of proteins to be shared between the datasets, excluding two proteins, a putative fimbriae usher associated with pilus assembly (StbC) and an outer membrane pore protein (OmpN) unique to the secretome (Figure 1B). These data support both an intracellular and extracellular role for the majority of the secreted proteins. An analysis of the cellular proteome demonstrates overlap among the limited and replete conditions with 50 proteins unique to iron-limited media and 87 proteins produced in the presence of iron (Figure 1C). For the extracellular environment, 121 proteins are specifically secreted in the absence of iron, and 211 proteins are associated with iron availability (Figure 1D). Across differential iron concentrations in both the cellular proteome and secretome samples (focusing on significantly different proteins in the respective comparisons), up to 50% of the proteins identified were associated with iron, including transporters, receptors, enzymes, siderophores, and virulence factors (Figure 1E). Taken together, our proteomic analysis enables definition of specific proteins and protein networks changing during nutrient limitation and reports global changes to the intracellular and extracellular environments during iron limitation.

### Iron Availability Differentially Alters the Cellular Proteome of *K. pneumoniae*

**FIGURE 2:**
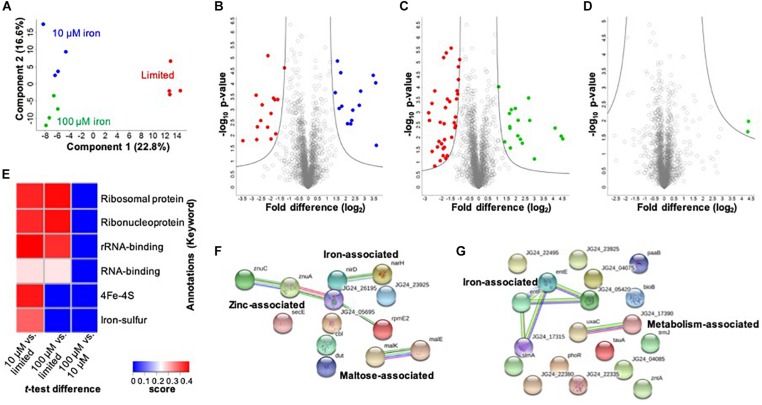
Profiling the impact of iron availability on the cellular proteome of *K. pneumoniae*. **(A)** Principal component analysis; clustering based on treatments. **(B)** Volcano plot depicting all proteins identified in limited and replete (10 μM iron) conditions, highlighting proteins with significant increases or decreases in abundance during 10 μM iron-replete (blue) vs. iron-limited (red) conditions. **(C)** Volcano plot depicting all proteins identified in limited and replete (100 μM iron) conditions, highlighting proteins with significant increases or decreases in abundance during 100 μM iron replete (green) vs. limited (red) conditions. **(D)** Volcano plot depicting all proteins identified in replete (10 and 100 μM iron) conditions, highlighting proteins with a significant increase in abundance during 100 μM iron-replete (green) conditions. **(E)** 1D annotation enrichment based on Uniprot Keywords [*p* < 0.05; false discovery rate (FDR) = 0.05; score > −0.5 < 0.5]. **(F)** STRING network analysis of significantly different proteins with an increase in abundance during replete vs. limited conditions. Protein clusters highlighted. **(G)** STRING network analysis of significantly different proteins with a decrease in abundance during replete vs. limited conditions. Protein clusters highlighted. For volcano plots: Student’s *t-*test, *p*-value ≤ 0.05; FDR = 0.05, S0 = 1.

In the cellular proteome, we identify 2,291 bacterial cellular proteins, and upon valid value filtering (protein must be identified in three out of four replicates, in at least one group), we pursue further analysis of 2,074 proteins. A PCA indicates clear clustering between limited and replete conditions as the largest component of distinction (component 1, 22.8%) and a second component displaying a distinction between the replete conditions (10 vs. 100 μM iron) (component 2, 16.6%) (Figure 2A). Biological replicate reproducibility was 97.3–98.1%, demonstrating high reproducibility among the cellular samples (Supplementary Figure S1).

Next, we define proteins with significant changes in abundance during iron limitation. Upon comparison of the limited vs. replete (10 μM) conditions, we identify 29 proteins with a significant change in abundance: 14 proteins significantly increased, and 15 proteins significantly decreased relative to the replete condition (Figure 2B and Supplementary Table S2). Proteins with higher abundance in the presence of 10 μM iron include those associated with iron binding (e.g., nitrate/nitrite reductases, NarG, NirB, NarH, NarJ, NirD), enzymes (e.g., phosphogluconate dehydratase, Edd), maltose import (e.g., maltoporin, LamB2), and virulence (e.g., lipid A palmitoyltransferase, PagP). Notably, the protein displaying the highest increase in abundance under replete conditions was a transcriptional regulator (i.e., bacterial regulatory protein, LysR), confirming the role of LysR as a global regulator of iron homeostasis in bacteria (Arafah et al., 2009; Zappa and Bauer, 2013; Chen et al., 2018). Conversely, proteins higher in the absence of iron (i.e., limited conditions) include those associated with iron binding (e.g., ferrioxamine receptor, KPN_02304), siderophores (e.g., 2,3-dihydroxybenzoate-AMP ligase, EntE, and EntF, EntC, EntB), metal transport (e.g., taurine transport protein, TauC), as well as enzymes (e.g., monooxygenase, KPN_01858), and adherence and virulence (e.g., lipoprotein, YajI). Our results demonstrate the diverse and significant roles that iron plays on regulation of the cellular proteome in *K. pneumoniae* and highlights our ability to quantify protein-level changes in cellular processes for acquisition of nutrients and bacterial adaptation within a nutrient-limited environment.

A comparison between limited vs. replete–excess (100 μM iron) conditions identifies 53 significantly different proteins with 18 proteins increased in abundance and 35 proteins decreased in abundance relative to the excess conditions (Figure 2C and Supplementary Table S3). Among these proteins, we detect overlap with the comparison between limited and 10 μM replete conditions, demonstrating a baseline or inherent response to iron availability, regardless of concentration. For example, PagP, LysR, and several maltose-binding proteins and enzymes were common among the replete conditions, whereas siderophore-associated proteins (e.g., EntF, EntC, EntE, and EntB) and iron transporters and receptors (e.g., KPN_02304) were common among the limited conditions. In consideration of unique proteins showing significant changes in abundance under replete–excess conditions vs. limited, multiple kinases (e.g., histidine kinase, PhoR), bacterial regulatory protein (e.g., DeoR), and enzymes (e.g., acid phosphatase, PhoC) were more abundant in the absence of iron.

Finally, we analyze differences in protein abundance only in consideration of available iron (i.e., replete conditions 10 vs. 100 μM) and identify two proteins with significant increases in abundance under replete–excess (100 μM) conditions, including a ribosomal protein and the uncharacterized protein (KPN_04244) (Figure 2D). Notably, the uncharacterized protein (KPN_04244), with potential roles in zinc binding, was also the most abundant protein in the presence of 100 μM iron when compared to iron limited conditions, supporting its role in iron homeostasis and providing justification for future investigation. Taken together, the cellular proteome profiling differentiates the bacterial response to changing iron availability and demonstrates the diverse impact on global processes within the cell.

To evaluate enrichment of categories in our dataset, we perform 1D annotation enrichment based on Uniprot-designated keywords (Figure 2E). A 1D annotation enrichment tests for every annotation term whether the corresponding numerical values have a preference to be systematically larger or smaller than the global distribution of the values for all proteins (Cox and Mann, 2012). Here, we observe a positive enrichment of ribosomal, ribonucleoprotein, and RNA-binding categories in both replete conditions relative to the limited conditions and an enrichment of iron-associated categories (e.g., 4Fe–4S and iron–sulfur clusters) in the presence of 10 μM iron compared to the limited conditions. Notably, a negative enrichment was observed for all categories in the replete (100 μM) condition compared to the lower replete (10 μM) condition. These results highlight the enrichment of iron-associated proteins during low iron conditions and emphasize an iron concentration-dependent cellular response.

Lastly, we aim to define networks of proteins altered under low iron conditions. For this analysis, we use the STRING database to visualize proteins showing a significant increase in abundance under replete (10 and 100 μM) conditions. This analysis highlights three clusters demonstrating a connection among nitrite and nitrate reductase (i.e., iron associated), zinc transporters, zinc-binding lipoprotein, and a ribosomal protein (i.e., zinc associated), and maltose import and transport (e.g., maltose associated) (Figure 2F). Next, we profile connections relevant to limited conditions and observe two clusters: iron associated (e.g., EntE, EntF, iron receptor, and siderophores) and metabolism associated (e.g., isomerase and dehydratase) (Figure 2G). All other proteins do not show connections within the parameters of the STRING database (i.e., known interactions, predicted interactions, and other) for detailed network mapping.

### Profiling of the Extracellular Environment During Iron Limitation Supports the Use of Multiple Methods for Protein Secretion

**FIGURE 3:**
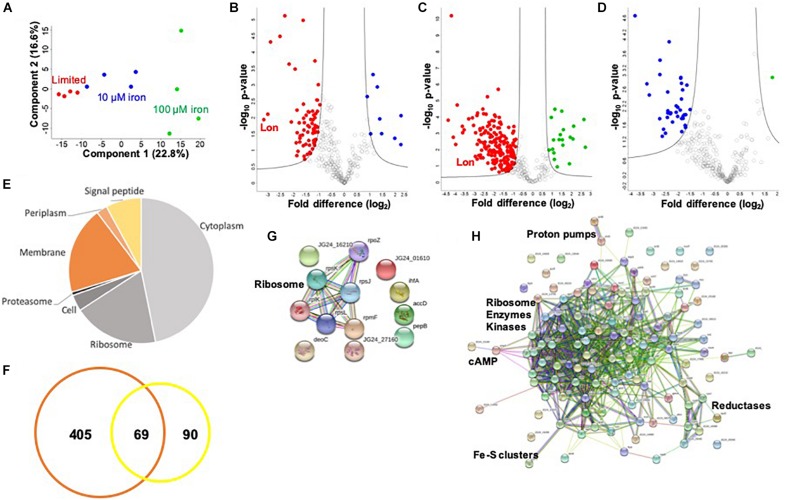
Secretome profiling of *K. pneumoniae* under iron limitation. **(A)** Principal component analysis; clustering based on treatments (component 1) and biological reproducibility (component 2). **(B)** Volcano plot depicting all proteins identified in limited and replete (10 μM iron) conditions, highlighting proteins with significant increases or decreases in abundance during 10 μM iron-replete (blue) vs. iron-limited (red) conditions. Lon protease demonstrated the largest decrease in abundance relative to the replete conditions (>8-fold) (highlighted). **(C)** Volcano plot depicting all proteins identified in limited and replete (100 μM iron) conditions, highlighting proteins with significant increases or decreases in abundance during 100 μM iron-replete (green) vs. iron-limited (red) conditions. Lon protease is highlighted. **(D)** Volcano plot depicting all proteins identified in replete (10 and 100 μM iron) conditions, highlighting proteins with significant increases or decreases in abundance during 100 μM (green) vs. 10 μM (blue) iron conditions. **(E)** Pie chart illustrating cellular compartments and signal peptides (undesignated by cellular compartment, but contain signal peptide) of secreted proteins. **(F)** Venn diagram of *K. pneumoniae* proteins identified in this secretome study (orange) vs. proteins identified in previous profiling of outer membrane vesicles (yellow). **(G)** STRING network analysis of significantly different proteins with an increase in abundance during replete vs. limited conditions. Protein clusters highlighted. **(H)** STRING network analysis of significantly different proteins with a decrease in abundance during replete vs. limited conditions. Protein clusters highlighted. For volcano plots: Student’s *t-*test, *p*-value ≤ 0.05; FDR = 0.05, S0 = 1.

To investigate a potential connection between iron availability and the extracellular environment of *K. pneumoniae*, we profile the secretome under limited and replete (10 and 100 μM iron) conditions. We identify 474 proteins in the media supernatant across the three conditions, and upon valid value filtering (protein must be identified in three out of four replicates, in at least one group), we select 305 proteins for further analysis. Biological replicate reproducibility is 76.0–94.3%, with variability increasing relative to the abundance of iron (Supplementary Figure S2). Here, a PCA plot shows the transition from limited to replete conditions as the largest component of distinction (component 1, 49.6%), and the second component relates to replicate reproducibility (component 2, 12.1%) (Figure 3A).

To identify proteins with significant changes in protein abundance in the extracellular environment of *K. pneumoniae* under different iron concentrations, we compare the secretome of replete (10 μM iron) to limited conditions and define 77 significantly different proteins (Figure 3B and Supplementary Table S4). Of these, nine proteins show a significant increase in abundance, including ribosomal and DNA-binding proteins (e.g., RpsJ, RpsL, and Hns), as well as metal-ion-binding proteins (e.g., peptidase B, PepB), an integration host factor subunit associated with recombination (e.g., IhfA), and an uncharacterized protein associated with stress response (e.g., YjbJ). Conversely, 68 proteins are higher in abundance under limited conditions, including many metal-ion-binding proteins (e.g., glutaredoxin, YdhD), transporters (e.g., ABC transport system, KPN_00582), virulence-associated proteins (e.g., capsule export protein, Wza and superoxide dismutase, SodB), kinases (e.g., pyruvate kinase, PykF), and the most highly abundant protein during low iron availability, Lon protease. The role of Lon protease in the extracellular environment has not been previously reported. However, we observe several known Lon protease network proteins and degradation targets in the secretome, including SecB, RpoB, and ClpP, supporting its presence outside of the cell during limited iron availability. Taken together, these data support the secretion of iron-regulating proteins into the extracellular environment during limiting conditions and demonstrate a novel connection between iron homeostasis and Lon protease production in *K. pneumoniae*.

Finally, we profile changes in the secretome under replete (100 μM) and limited iron availability. Here, we identify 205 significantly different proteins; 20 proteins show a significant increase in abundance under replete conditions, and 185 proteins are significantly higher in the absence of iron (limited) (Figure 3C and Supplementary Table S5). Similar to our findings in the cellular proteome, we observe an overlap with the secretome profile at 10 μM iron. For example, ribosomal and DNA-binding proteins (e.g., RpsJ, HupB) are common under replete conditions regardless of iron concentration, as well as an integration host factor (e.g., IhfA) and peptidase (e.g., PepB). Notably, 61 proteins are common under limited conditions, including metal ion-binding proteins (e.g., transketolase, TktA), capsule-associated proteins (e.g., Wza), and proteases (e.g., Lon and ClpP). We also observe differences in protein abundance in the presence of iron-replete conditions (10 vs. 100 μM) and identify 37 proteins with an increased abundance at 10 μM and one protein with an increased abundance at 100 μM (Figure 3D and Supplementary Table S6). These unique proteins include a lipoprotein (e.g., KPN_02168), export protein (e.g., SecB), and enzymes (e.g., dehydrogenase flavoprotein, SdhA). These data support a baseline release or secretion of proteins into the extracellular environment of *K. pneumoniae* in the presence of iron in a concentration-independent manner and substantiates the influence of iron on the release of Lon protease.

Notably, of the proteins present in the extracellular environment, 30% contain a signal peptide, transmembrane domain, or associated with the membrane or extracellular compartments according to gene ontology cellular compartments classification (Figure 3E). This analysis categorizes conventionally secreted proteins but does not identify proteins that undergo non-conventional methods of secretion, including proteins secreted or released from the cell through “guilt-by-association” (i.e., binding or attachment to secreted proteins). In addition, our secretome profiles may also include proteins released in vesicles, which explain the presence of classically intracellular proteins (e.g., ribosomes) in the extracellular environment. For example, previous proteomic profiling of *K. pneumoniae* outer membrane vesicles (OMVs) identified 159 proteins with predicted localization in the extracellular space (*n* = 13), outer membrane (*n* = 24), periplasmic space (*n* = 25), inner membrane (*n* = 13), and cytoplasm (*n* = 84) (Lee et al., 2012). Direct comparison of our secretome dataset with the OMV proteins demonstrates overlap of 69 proteins, including several ribosomal proteins and elongation factors (e.g., RpsT, Tsf), chaperones (e.g., HtpG, GroL), lipoproteins (e.g., Lpp), kinases (e.g., nucleoside diphosphate kinase, Ndk), and proteases (e.g., serine endoprotease, DegP) (Figure 3F). We also quantify changes in protein abundance and found an overall increase in vesicle-associated proteins under limited vs. replete iron conditions (Supplementary Figure S3). This comparison supports our identification of proteins in the extracellular environment through conventional, non-conventional, and vesicle-associated roles in secretion and supports an increase in vesicle-associated proteins under low iron conditions.

Lastly, we define networks of proteins altered under low iron conditions in the extracellular environment. For this analysis, we use the STRING database to analyze proteins showing a significant increase in abundance during replete conditions and observe a small network of ribosomal proteins (e.g., RplK), which may be linked to the presence of vesicles in the media (Figure 3G). Analysis of proteins elevated under limited conditions highlights a dominant cluster of proteins, including ribosomal (e.g., RpsP), enzymes (e.g., isomerase, Pgi), and kinases (e.g., PyrH) (Figure 3H). Peripheral protein networks also support connections to reductases (e.g., CysH), proton pumps (e.g., PntA/B), cyclic AMP receptor (e.g., membrane protein, YchH), and iron–sulfur cluster assembly (e.g., cysteine desulfurase, IscS). All other proteins did not show connections within the parameters of the STRING database (i.e., known interactions, predicted interactions, and other) for detailed network mapping. Taken together, our profiling of the *K. pneumoniae* secretome highlights common and unique proteins and protein networks important during iron limitation and supports previous findings of vesicle-associated proteins in *K. pneumoniae*, while it suggests novel roles for highly conserved enzymes in the extracellular environment.

### Phenotypic Profiling of Δ*lon* Supports a Role for Lon Protease in Bacterial Growth, Cell Division, Iron Utilization, and Virulence

**FIGURE 4:**
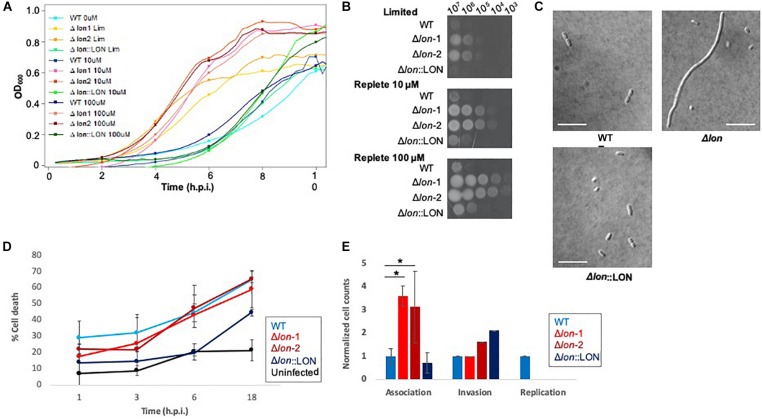
Phenotypic profiling of Δ*lon* highlights the diverse impact of Lon protease in *K. pneumoniae.* **(A)** Growth curve of WT, Δ*lon*:LON, and Δ*lon* strains under limited (LIM), 10 μM iron-replete, and 100 μM iron-replete conditions. OD_600_ measured; error bars represent standard deviation. Experiment performed in biological triplicate, technical duplicate with two independent mutant strains. **(B)** Iron utilization plates of WT, Δ*lon*:LON, and Δ*lon* strains under limited (LIM), 10 μM iron-replete, and 100 μM iron-replete conditions. Images captured at 24 h; dilution series from 10^7^ to 10^2^. **(C)** Differential interference contrast microscopy at 100 × for WT, Δ*lon*:LON, and Δ*lon* strains under 100 μM iron-replete conditions. Scale bar = 10 μm. **(D)** Lactate dehydrogenase (LDH) assay of immortalized BALB/c macrophages infected (MOI 50:1) with *K. pneumoniae* WT, Δ*lon*:LON, and Δ*lon* strains during the time course of infection. Error bars represent standard deviation. **(E)** Association, invasion, and replication assay highlighting differences among the WT, Δ*lon*:LON, and Δ*lon* strains during infection. *Student’s *t-*test *p*-value ≤ 0.05.

Given our detection of Lon protease with a >8-fold increase in the extracellular environment of *K. pneumoniae* under low iron availability, we hypothesize a role in iron homeostasis and evaluate the impact of Lon on bacterial growth and virulence during nutrient limitation. First, using lon deletion mutant (Δ*lon*), WT, and Δ*lon*:LON *K. pneumoniae* strains, we perform a growth curve in limited and replete (10 and 100 μM iron) media. In all strains, we observe a decrease in growth in the limited media relative to replete conditions, with both iron concentrations demonstrating higher rates of growth (Figure 4A and Supplementary Figure S4). The Δ*lon* strain grew at a faster rate than the WT under all conditions and demonstrates an increase in growth under replete conditions. We observe similar findings with iron utilization plates, supporting the differential growth of Δ*lon* and WT (and Δ*lon*:LON) strains (Figure 4B). Taken together, these results support the role of Lon protease in cellular regulation and homeostasis during bacterial growth and proliferation. Next, we collect samples for microscopy at early stationary phase under the different growth conditions. Although we do not observe differences in bacterial morphology influenced by iron availability, we report an elongated, filamented phenotype of the Δ*lon* strain, which was rescued in the Δ*lon*:LON strain (Figure 4C). These results suggest an accumulation of cell-division-associated proteins in the absence of Lon protease in *K. pneumoniae*.

To evaluate the impact of Lon protease on bacterial virulence, we performed a macrophage infection assay and determine rates of host cell death associated with the release of lactate dehydrogenase (LDH). We observe an increase in LDH release in the WT strains at 1 and 3 hpi, compared to the Δ*lon* strains, supporting a role for Lon protease in virulence of *K. pneumoniae* (Figure 4D). By 6 hpi, WT and Δ*lon* strains show similar cell death (ranging from 40 to 50%) and remaining consistent until 18 hpi (60%). Uninfected macrophage cells show consistent rates of LDH release (∼2%) from 1 to 6 h, with an increase to 25% after 18 h, values likely associated with natural cell death rates during *in vitro* culturing. Similarly, the Δ*lon*:LON strain shows similar trends in macrophage cell death as WT. Given the differences in growth rates between the WT and Δ*lon* strains, the variances in macrophage cell death could be attributed to these growth patterns. To evaluate this hypothesis, we perform an association, invasion, and replication/survival assay (Wu et al., 2014). Here, we observe a significant increase in association of the Δ*lon* strains compared to WT at 0 hpi but similar rates of invasion among the strains and the ability of WT to survive within macrophage after 24 hpi (Figure 4E).

### Novel Degradation Targets of Lon Protease Support New Mechanisms for Iron Homeostasis

**FIGURE 5:**
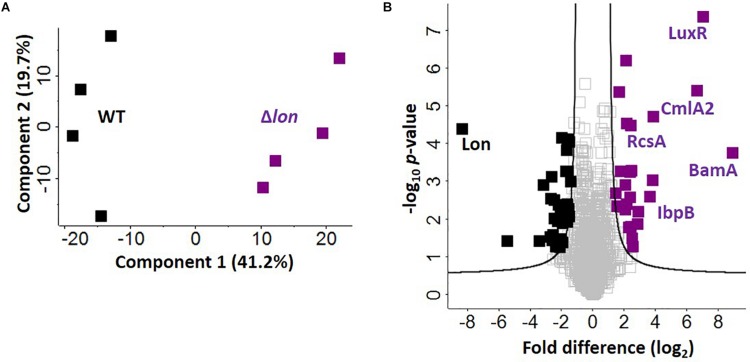
Defining candidate degradation products of Lon protease in *K. pneumoniae*. **(A)** Principal component analysis; clustering based on strain. **(B)** Volcano plot depicting all proteins identified during cellular proteome profiling of *K. pneumoniae* wild-type (WT) and Δ*lon* strains. Proteins showing an increase in abundance in the absence of Lon suggest targets of degradation (purple) compared to proteins unchanged (gray) or with a higher abundance in the WT (black). Known interactors (i.e., RcsA, IbpB, and LuxR are highlighted), as well as proteins with highest fold-change differences (i.e., BamA and CmlA2). Student’s *t-*test, *p*-value ≤ 0.05; false discovery rate (FDR) = 0.05, S0 = 1.

Given the presence of Lon protease in the extracellular environment under iron limited conditions, we hypothesize a role for Lon protease in the regulation (e.g., degradation) of iron-associated proteins. To evaluate this, we perform a quantitative proteomic analysis on the cellular proteome of the WT and Δ*lon* strains. This comparison allows us to identify proteins that accumulate in the mutant strain, suggesting possible targets of Lon degradation. We identify 1,743 proteins (after filtering for proteins present in three of four replicates), which cluster based on presence or absence of Lon protease (component 1, 41.2%) (Figure 5A). Based on an FDR-corrected *p*-value, we detect 59 proteins with significant changes in protein abundance associated with Lon protease production (Figure 5B). Specifically, we are interested in proteins accumulating in the absence of Lon (Table 1). Our proteomic profiling confirms identification of known Lon protease targets: RcsA, a transcriptional regulatory protein; LuxR, a bacterial regulatory protein; and IbpB, a small heat shock protein, validating our approach to discover Lon protease degradation targets (Torres-Cabassa and Gottesman, 1987; Manukhov et al., 2006; Bissonnette et al., 2010). We also report proteins with connections to previously defined degradation targets of Lon protease, including three transcriptional regulators, i.e., YiaG (putative HTH-type transcriptional regulator), LrhA (NADH dehydrogenase transcriptional regulator), and YchA (putative transcriptional regulator), as well as two enzymes, i.e., Gsk (inosine–guanosine kinase) and YdcP (putative protease) (Gibson and Silhavy, 1999; Keeney et al., 2008; Chiang et al., 2011; Osbourne et al., 2014). Taken together, these results confirm our ability to accurately profile the impact of Lon protease on the *K. pneumoniae* proteome.

**TABLE 1:** Accumulated proteins in absence of Lon protease in *K. pneumoniae*.

**Uniprot**	**Gene**	**Protein name**	**Relation to Lon protease**	**Difference (log_2_)**
**Transcriptional regulation; DNA/RNA binding**				
A6TDV9	luxR	Putative bacterial regulatory protein	Known	7.02
A6TC04	truA	tRNA pseudouridine synthase A	Novel	2.44
A6TB73	rcsA	Transcriptional regulatory protein RcsA	Known	2.42
A6TFH0	yiaG	Putative HTH-type transcriptional regulator	Connection	2.37
A6TBX5	lrhA	NADH dehydrogenase transcriptional regulator, LysR family	Connection	2.30
A6TGE7	fabR	HTH-type transcriptional repressor	Novel	2.07
A6TAN6	ychA	Transcriptional regulator	Connection	2.06
A6TI69	KPN_05911	Uncharacterized	Novel	1.75
**Iron-associated**				
A6TC41	yfeC	Putative negative regulator	Novel	3.81
A6TC91	aegA	Putative oxidoreductase, Fe–S subunit	Novel	2.14
A6THK0	yjgB	Putative alcohol dehydrogenase	Novel	2.12
A6TD48	cysI	Sulfite reductase [NADPH] hemoprotein beta-component	Novel	1.70
**Enzymatic activity**				
A6THT3	KPN_04771	Putative transposase	Novel	3.87
A6TET8	smg	Uncharacterized	Novel	2.91
A6TF04	codA	Putative cytosine deaminase	Novel	2.87
A6T5N8	gsk	Inosine–guanosine kinase	Connection	2.55
A6T9U4	ydcP	Putative protease	Connection	2.52
A6TFY4	KPN_04085	Putative glycoside hydrolase	Novel	2.47
A6T7R2	KPN_01200	HNHc domain-containing protein	Novel	2.36
A6TAL7	narJ	Nitrate reductase 1	Novel	2.17
A6THH9	yjgM	Putative acyltransferase	Novel	1.55
A6TEH7	yhbU	Putative collagenase	Novel	1.48
**Transport & Stress response**				
A6TGX3	bamA	Outer membrane protein assembly factor BamA	Novel	8.91
A6TJ41	cmlA2	Chloramphenicol acetyltransferase	Novel	6.65
A6TFY9	ibpB	Small heat shock protein	Known	3.62
A6TH35	KPN_04514	Uncharacterized	Novel	1.91

Finally, we report novel candidate degradation products of Lon protease and observe a direct connection between iron homeostasis and Lon activity. These proteins cover a broad range of cellular processes, including transcriptional regulation and DNA/RNA binding (e.g., HTH-type transcriptional regulator, FabR), iron-associated proteins (e.g., putative oxidoreductase, AegA), enzymes (e.g., putative transposase, KPN_04771 and putative collagenase, YhbU), and transport and stress response proteins (e.g., outer membrane protein assembly factor, BamA). Of the 26 accumulated proteins, three are membrane associated (e.g., YfeC, KPN_04085, BamA) and two proteins contain signal peptides (e.g., BamA and KPN_04514). These data provide further evidence of a role for Lon protease in the extracellular environment of *K. pneumoniae* during iron limitation. We also note the presence of CmlA2, a chloramphenicol acetyltransferase with a significant increase in abundance in the Δ*lon*; this is likely a result of chloramphenicol selection for the mutant strain.

Notably, in the WT strain, Lon protease demonstrates the greatest difference relative to Δ*lon*, confirming its proper deletion in the mutant strain. We also observe 33 proteins showing a significant reduction in abundance in the Δ*lon* strain, suggesting a role for Lon protease in cellular homeostasis through negative feedback loops (e.g., quorum sensing) or redundant mechanisms (e.g., overlap of a protein degradation function with ClpP or other proteases and peptidases) within the bacterial cell to remove abnormal proteins (Supplementary Table S7).

## Discussion

In this study, we use quantitative proteomics to profile the global impact of iron limitation on the cellular proteome and extracellular environment (secretome) of *K. pneumoniae*. Our data reveal the deepest proteome to date of *K. pneumoniae*, reporting reprogramming of processes associated with transcriptional regulation and iron acquisition, transport, and binding (i.e., siderophores) in the cellular proteome. These findings highlight our ability to comprehensively, and from an unbiased perspective, profile proteins directly and indirectly involved in maintaining iron homeostasis within a bacterial system. We confirm the presence of proteins in the extracellular environment through association with conventional and non-conventional modes of secretion, as well as vesicle release, and we demonstrate a new role for Lon protease in promoting iron homeostasis outside the cell. With this information, we characterize the role of Lon protease in *K. pneumoniae* related to bacterial growth, cell division, and virulence, and we uncover novel degradation candidates and relate accumulating protein abundance to improved iron utilization strategies in the absence of the protease. Overall, our work demonstrates a new role for Lon protease in the extracellular environment during nutrient limitation, proposes degradation candidates not previously defined in other bacterial systems, and provides evidence of novel connections between Lon and iron homeostasis.

In a limited environment (e.g., nutrient limited media, host system), many microorganisms, including Gram-negative bacteria, secrete siderophores, small high-affinity iron-chelating molecules, for acquisition of iron (Holden and Bachman, 2015). These factors are critical for virulence and, during infection, induce secretion of interleukin-6, CXCl1, and CXCL2, assist with bacterial dissemination to the spleen, and stabilize hypoxia inducible factor-1a (HIF-1a) (Holden et al., 2016). An example of siderophores in bacteria are enterobactin (Ent), a prototypic catecholate siderophore that effectively outcompetes host iron-binding proteins in a limited environment (Crumbliss and Harrington, 2009). In agreement with previous reports, our quantitative proteomic analysis confirms the role of siderophores in iron acquisition in *K. pneumoniae* through increased production of Ent proteins (e.g., entF, entC, entE, and entB) under limited conditions. We also observe a > 6-fold increase in abundance of CirA (an outer membrane pore protein) associated with TonB (an energy-transducing system critical for siderophore secretion) under limited conditions. These data support the rapid and powerful response of *K. pneumoniae* during iron limitation through production of siderophores (Noinaj et al., 2010). We did not observe siderophores in the extracellular environment of *K. pneumoniae* during iron limitation; this is likely attributed to the timing of sample collection (i.e., mid-log phase), as previous detection of Ent in the supernatant was strain specific and detected at stationary phase (following overnight growth) (Holden et al., 2018). However, our secretome profiling did report the release of numerous iron- and metal ion-binding proteins during nutrient limitation, including cluster assembly proteins (e.g., glutaredoxin) and transporters (e.g., ABC transport system). Taken together, our ability to detect siderophore-associated proteins in the cellular environment, prior to secretion, demonstrates the dynamics of iron homeostasis in *K. pneumoniae* and supports our ability to profile mechanisms of cellular response to iron limitation in multiple environments.

To adapt to diverse stresses in the environment, including nutrient limitation, bacteria have evolved sophisticated mechanisms in response to external stimuli. The profile of the bacterial proteome is modified through transcriptional regulation and protein degradation mediated by intracellular proteases (Gottesman, 1996). For example, Lon protease is classically defined as an ATP-dependent cytoplasmic serine protease responsible for degradation of misfolded proteins. However, in this study, we report the presence of Lon protease within the extracellular environment of *K. pneumoniae* in response to low iron. Despite the classical role of Lon as an intracellular protease, its significant increase in abundance (>8-fold) outside of the cell, along with the identification of several Lon-associated proteins and degradation targets in the culture supernatant, including (i) secB, a chaperone involved in the quality control of presecretory proteins; (ii) clpP, a highly conserved caseinolytic protease involved in proteolysis of damaged and misfolded proteins; and (iii) rpoB, a DNA-directed RNA polymerase subunit and target of Lon, support the presence of the protease in the extracellular environment (Flynn et al., 2003; Takaya et al., 2008). We predict that the presence of Lon protease in the extracellular environment may play roles in degrading host proteins to release iron or other scarce metal ions into the surrounding environment. Given the substrate selectivity by multiple domains of bacterial and human Lon proteases, the enzyme’s release by the bacteria may evolve to recognize host degradation targets as a mode of virulence (Karlowicz et al., 2017; He et al., 2018). We also propose that Lon protease may be degrading host proteins that are detrimental to the bacteria or degrading host proteins to promote invasion of the pathogen. Recent discoveries of new Lon protease targets and novel roles for the enzyme in host cell maintenance suggest a plethora of modes of action for Lon protease not previously explored (Bezawork-Geleta et al., 2015; Pinti et al., 2016; Zhou et al., 2018). These potential roles for Lon protease in the extracellular environment are very intriguing and warrant further study. Moreover, our detection of proteins undergoing both conventional (e.g., signal peptide, transmembrane domain) and non-conventional (e.g., membrane-associated, guilt-by-association) modes of secretion, along with overlap between the secretome and OMV profiles, and the presence of proteases in extracellular bacterial vesicles further supports the presence of Lon protease in the extracellular environment (Dalbey et al., 2012; Lee et al., 2012; Cezairliyan and Ausubel, 2017; Kim et al., 2018). We also compare the abundance profiles of vesicle-associated *K. pneumoniae* proteins and observe an overall increase in vesicle-associated proteins under limited vs. replete iron conditions. These results coincide with previous reports of increased vesicle production by bacterial pathogens under nutrient-limiting environments (Prados-Rosales et al., 2014; Orench-Rivera and Kuehn, 2016; Roier et al., 2016).

Our observation of an increased growth rate of Δ*lon* strain compared to WT in the presence or absence of iron is supported by our proteomic data showing accumulation of transcriptional and cell division regulators, which may lead to increased cell proliferation and turnover rates, resulting in higher growth. In addition, previous reports have observed a higher growth rate in the absence of Lon under nutrient-rich and limited conditions, as well as a reduction in cell growth when Lon is overexpressed (Goff and Goldberg, 1987; Nicoloff and Andersson, 2013). We also observe an increase in growth rate for the Δ*lon* strain in the presence of iron, compared to limited conditions. This difference may be attributed to the presence of higher iron levels for improved viability of the cells. Alternatively, given our observation of accumulation of iron-associated proteins in the absence of Lon by proteomic profiling, we predict that the increased growth rate is due to higher uptake of iron from the surrounding environment by the deletion strain. This notion is supported by previously defined roles for the identified iron-associated proteins. For example, YfeC, a chelated iron transport system membrane protein in *Yersinia pestis*, demonstrates a role in iron acquisition, and its accumulation in the absence of Lon supports increased uptake of iron and improved bacterial growth rates (Bearden et al., 1998). The other proteins, AegA and CysT, are connected to metal binding through iron–sulfur clusters as defined by Gene Ontology Molecular Function profiling, or the use of metal cations for phosphatase activity (e.g., YigB). Taken together, our observation of differences in growth rate of the Δ*lon* strain compared to WT corresponds with previous studies and provides a novel connection between accumulation of iron-associated proteins and growth in the absence of the enzyme.

In the absence of Lon protease, we observe a moderately lower rate of macrophage cell death compared to the WT, suggesting that the absence of Lon contributes to reduced virulence. A connection between Lon and virulence has been previously reported (Breidenstein et al., 2012b; Rogers et al., 2016; Culp and Wright, 2017). To explore a reason for the variance in cell death and whether it was attributed to the different growth rates of Δ*lon* vs. WT, we assessed cell counts during association, invasion, and replication of macrophages. Our results show that immediately following co-culture, the deletion strain associates with macrophage at a significantly higher rate than the WT strain; however, this difference is not maintained during invasion and replication. Such a phenotype for Lon protease mutants has been observed in *Salmonella* Typhimurium during assessment of invasion and survival in epithelial cells and murine macrophage (Takaya et al., 2002, 2003). We hypothesize that given the reduced ability of Δ*lon* to properly divide, resulting in elongated cells, macrophages are unable to phagocytose the elongated cells at the same rate as WT. Furthermore, the Δ*lon* shows an impaired ability to survive within the macrophage, supporting the ability of macrophage to clear mutant bacterial cells within 24 h of exposure.

## Conclusion

In conclusion, using state-of-the-art mass-spectrometry-based quantitative proteomics and advanced bioinformatic platforms, we define the global impact of iron limitation on the cellular proteome and secretome of *K. pneumoniae*. We uncover new mechanisms associated with regulation of iron homeostasis across multiple cellular processes and report a novel role for the highly conserved Lon protease in the extracellular environment. We validate our findings with *in silico* characterization of cellular and secreted proteins, and we confirm the role of Lon protease in bacterial growth, cell division, iron utilization, and virulence. We also uncover novel degradation candidates of Lon protease with diverse roles in transcriptional regulation, iron homeostasis, enzymatic activity, and transport. Overall, our analysis provides a comprehensive profile of the bacterial responses to nutrient limitation and provides evidence for new avenues of exploration to further our understanding of the diverse effects of Lon protease activity within microbial systems.

## Data Availability Statement

The mass spectrometry proteomics data have been deposited in the PRIDE partner repository for the ProteomeXchange Consortium with the data set identifier: http://www.ebi.ac.uk/pride/archive/projects/PXD015623.

## Author Contributions

JG-M and AS conceived the project and planned the experiments. BM, AS, and JY performed the experiments. BM, AS, JY, and JG-M performed the data analysis and interpretation and wrote and edited the manuscript. BM, AS, and JG-M generated the figures.

## Conflict of Interest

The authors declare that the research was conducted in the absence of any commercial or financial relationships that could be construed as a potential conflict of interest.

## Footnotes

1 http://www.uniprot.org

2 http://www.R-project.org/

3 https://string-db.org

## Supplementary Material

FIGURE S1 Cellular proteome column correlation heatmap for replicate reproducibility.

FIGURE S2 Secretome column correlation heatmap for replicate reproducibility.

FIGURE S3 Protein abundance of vesicle-associated proteins in the secretome.

FIGURE S4 Western blot of arabinose-inducible complement *K. pneumoniae* strain.

TABLE S1 Primer sequences for *K. pneumoniae* genetic manipulation.

TABLE S2 Significantly different proteins in cellular proteome under replete (10 μM) vs. limited conditions.

TABLE S3 Significantly different proteins in cellular proteome under replete (100 μM) vs. limited conditions.

TABLE S4 Significantly different proteins in secretome under replete (10 μM) vs. limited conditions.

TABLE S5 Significantly different proteins in secretome under replete (100 μM) vs. limited conditions.

TABLE S6 Significantly different proteins in secretome under replete (10 μM vs. 100 μM) conditions.

TABLE S7 Significantly different proteins in cellular proteome of Δ*lon* vs. WT strains.
