# Supplementary figures and images for: Iron Limitation in Klebsiella pneumoniae Defines New Roles for Lon Protease in Homeostasis and Degradation by Quantitative Proteomics
Source: Front Microbiol. 2020 Apr 24;11:546. doi: 10.3389/fmicb.2020.00546 (PMC7194016; doi:10.3389/fmicb.2020.00546)

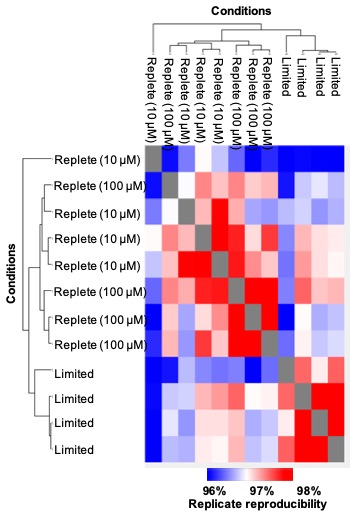

Supplement: FIGURE S1 — Cellular proteome column correlation heatmap for replicate reproducibility. [file Image_1.JPEG]

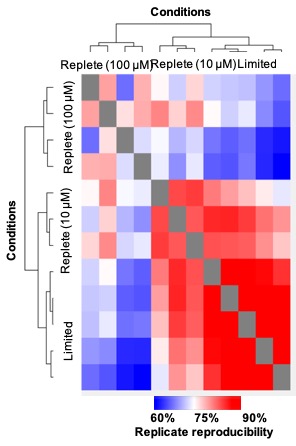

Supplement: FIGURE S2 — Secretome column correlation heatmap for replicate reproducibility. [file Image_2.JPEG]
