# Supplementary material for: Iron Limitation in Klebsiella pneumoniae Defines New Roles for Lon Protease in Homeostasis and Degradation by Quantitative Proteomics
Source: Front Microbiol. 2020 Apr 24;11:546. doi: 10.3389/fmicb.2020.00546 (PMC7194016; doi:10.3389/fmicb.2020.00546)
Supplement: FIGURE S3 — Protein abundance of vesicle-associated proteins in the secretome. [file Image_3.pdf]

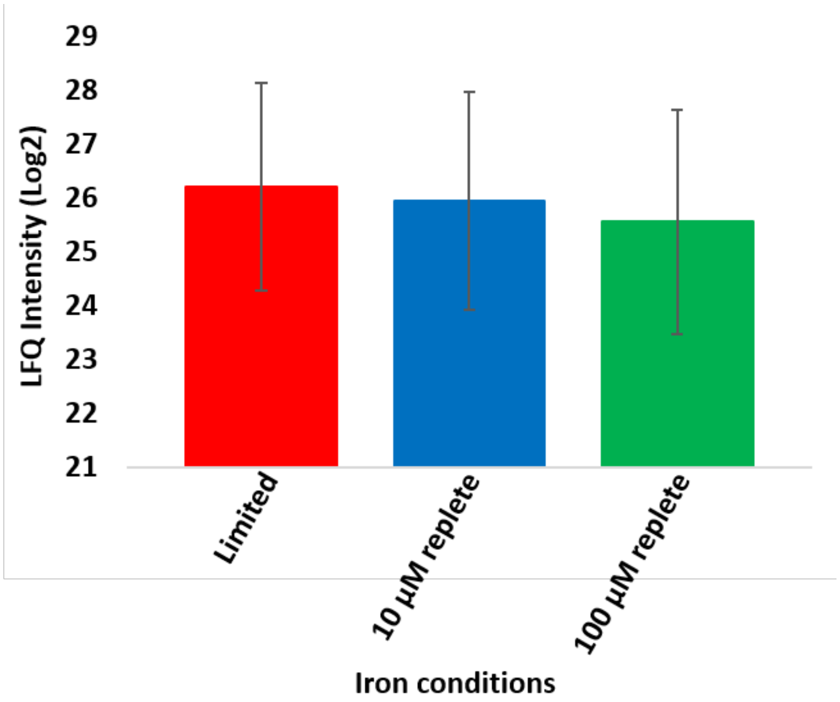

Supplemental Figure 3: Abundance of vesicle-associated proteins detected in the secretome of *K. pneumoniae*. Plot represents the label-free quantification (LFQ) abundance (log_2) of proteins common between the secretome of *K. pneumoniae* under changing iron conditions and previously profiled outer membrane vesicles. Average abundance reported, error bars represent standard deviation. Experiment performed in biological quadruplicate and technical duplicate.
