# Supplementary material for: Iron Limitation in Klebsiella pneumoniae Defines New Roles for Lon Protease in Homeostasis and Degradation by Quantitative Proteomics
Source: Front Microbiol. 2020 Apr 24;11:546. doi: 10.3389/fmicb.2020.00546 (PMC7194016; doi:10.3389/fmicb.2020.00546)
Supplement: FIGURE S4 — Western blot of arabinose-inducible complement K. pneumoniae strain. [file Image_4.pdf]

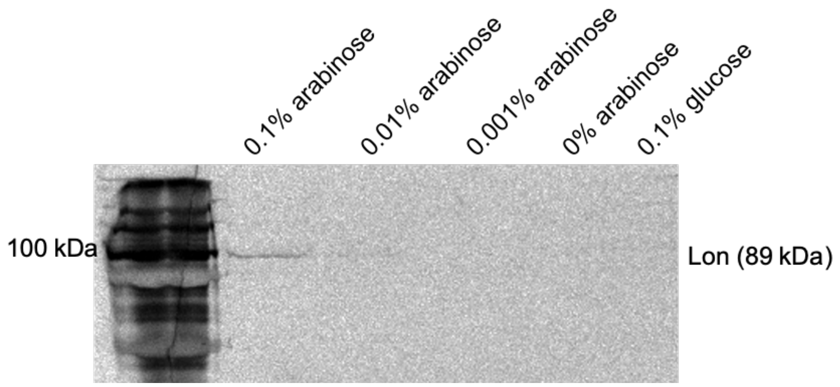

Supp. Figure 4: Western blot of arabinose-inducible Δ*lon*::LON *K. pneumoniae* strain. The Δ*lon* strain was transformed with a His-tagged LON protein under induction with arabinose. The western blot shows production of Lon protease (89 kDa) with 0.1% arabinose and a lower production at 0.01% arabinose. Lon protease production is undetectable with 0.001% arabinose, 0% arabinose, or 0.1% glucose, confirming production of the protein under the specified conditions. All subsequent experiments using the Δ*lon*::LON strain were performed in 0.1% arabinose.
